# Inherited mutations affecting the SRCAP complex are central in moderate-penetrance predisposition to uterine leiomyomas

**DOI:** 10.1016/j.ajhg.2023.01.009

**Published:** 2023-02-10

**Authors:** Niko Välimäki, Vilja Jokinen, Tatiana Cajuso, Heli Kuisma, Aurora Taira, Olivia Dagnaud, Sini Ilves, Jaana Kaukomaa, Annukka Pasanen, Kimmo Palin, Oskari Heikinheimo, Ralf Bützow, Lauri A. Aaltonen, Auli Karhu

**Affiliations:** 1 Department of Medical and Clinical Genetics, University of Helsinki, Helsinki, Finland; 2 Applied Tumor Genomics Research Program, Research Programs Unit, University of Helsinki, Helsinki, Finland; 3 Department of Pathology, University of Helsinki and Helsinki University Hospital, Helsinki, Finland; 4 iCAN Digital Precision Cancer Medicine Flagship, University of Helsinki, Helsinki, Finland; 5 Department of Obstetrics and Gynecology, University of Helsinki and Helsinki University Hospital, Helsinki, Finland

**Keywords:** uterine leiomyoma, medical genetics, tumor genetics, germline mutation, inherited mutation, SRCAP complex, H2A.Z histone variant, predisposition, UK Biobank, next-generation sequencing

## Abstract

Uterine leiomyomas (ULs) are benign smooth muscle tumors that are common in premenopausal women. Somatic alterations in *MED12*, *HMGA2*, *FH*, genes encoding subunits of the SRCAP complex, and genes involved in Cullin 3-RING E3 ligase neddylation are mutually exclusive UL drivers. Established predisposition genes explain only partially the estimated heritability of leiomyomas. Here, we examined loss-of-function variants across 18,899 genes in a cohort of 233,614 White European women, revealing variants in four genes encoding SRCAP complex subunits (*YEATS4*, *ZNHIT1*, *DMAP1*, and *ACTL6A*) with a significant association to ULs, and *YEATS4* and *ZNHIT1* strikingly rank first and second, respectively. Positive mutation status was also associated with younger age at diagnosis and hysterectomy. Moderate-penetrance UL risk was largely attributed to rare non-synonymous mutations affecting the SRCAP complex. To examine this disease phenotype more closely, we set out to identify inherited mutations affecting the SRCAP complex in our in-house sample collection of Finnish individuals with ULs (n = 860). We detected one individual with an *ACTL6A* splice-site mutation, two individuals with a *YEATS4* missense mutation, and four individuals with *DMAP1* mutations: one splice-site, one nonsense, and two missense variants. These individuals had large and/or multiple ULs, were often diagnosed at an early age, and many had family history of ULs. When a somatic second hit was found, *ACTL6A* and *DMAP1* were silenced in tumors by somatic mutation and *YEATS4* by promoter hypermethylation. Decreased H2A.Z staining was observed in the tumors, providing further evidence for the pathogenic nature of the germline mutations. Our results establish inactivation of genes encoding SRCAP complex subunits as a central contributor to moderate-penetrance UL predisposition.

## Teaser

UK Biobank whole-exome data revealed an association between mutations in genes encoding the SRCAP complex and uterine leiomyomas (ULs). In our collection of 860 individuals, we identified seven women with inherited mutations in *ACTL6A*, *YEATS4*, and *DMAP1*. Our results establish these genes as central contributors to moderate-penetrance UL predisposition.

## Introduction

Uterine leiomyomas (ULs [MIM: 150699]) are common benign smooth muscle tumors of the myometrium with a cumulative incidence of up to 70% in premenopausal women.^1^ ULs are often asymptomatic, but it has been estimated that about 25%–30% of individuals with ULs develop symptoms, including pelvic pain, abnormal uterine bleeding, and adverse effects on fertility.^1^^,^^2^ The only permanent treatment options are invasive, and ULs continue to be the leading cause of hysterectomy.^3^^,^^4^ The overall annual cost of ULs has been estimated to exceed the combined costs of breast cancer and colon cancer in the United States.^5^

Somatic hotspot mutations in *MED12*, chromosomal rearrangements of *HMGA2* leading to its overexpression, and loss of *FH* account for about 90% of ULs.^6^^,^^7^^,^^8^^,^^9^^,^^10^^,^^11^ ULs can be divided into distinct molecular subclasses based on these mutually exclusive genetic alterations. In a recently discovered UL subclass, mutations in *CUL3*, *NAE1*, *NEDD8*, and *UBE2M* were shown to disrupt neddylation of the Cullin 3-RING E3 ligase, leading to the NRF2 pathway activation.^12^ We also reported another UL subclass, characterized by somatic mutations in six out of nine genes encoding SRCAP complex subunits (*ACTL6A*, *DMAP1*, *SRCAP*, *VPS72*, *YEATS4*, and *ZNHIT1*). This complex is a key player in depositing variant histone H2A.Z into chromatin.^13^ The mutations were typically accompanied by inactivation of the other allele by genetic loss or promoter hypermethylation, resulting in defective H2A.Z deposition.^14^

Several lines of evidence indicate that genetic predisposition has a role in the genesis of UL. Previous studies have shown that the risk of developing UL is significantly higher for individuals with a positive family history.^15^^,^^16^ Twin studies and population differences in prevalence of UL support the role of genetic predisposition in their development.^15^^,^^17^^,^^18^ Genome-wide association studies (GWASs) have highlighted several low-risk UL predisposition loci.^19^^,^^20^^,^^21^^,^^22^^,^^23^ Germline mutations in *FH* predispose to uterine and skin leiomyomas with very high penetrance. In addition, the relative risk of aggressive early-onset kidney cancer is elevated (hereditary leiomyomatosis and renal cell cancer or HLRCC syndrome).^10^^,^^24^ Germline mutations in *PTEN* and *SMARCB1*, causing Cowden syndrome and schwannomatosis, respectively, are also suggested to increase risk of ULs, although the association is much less clear.^25^^,^^26^ Taken together, these GWASs and family studies provide insight into the low- and high-penetrance ends of the estimated heritability of UL, respectively, while the more challenging to approach moderate-risk UL predisposition has remained largely unexplored.

In our recent study focusing on somatic UL-driving events,^14^ we also discovered an association between the incidence of UL and hereditary loss-of-function (LoF) variants in two genes encoding SRCAP complex subunits, *YEATS4* and *ZNHIT1*. This finding led us to explore in the present study the relative contribution of pathogenic germline mutations affecting the SRCAP complex in the full release of UK Biobank (UKB) exomes and to search for germline mutations in genes encoding SRCAP complex subunits in our sample collection of Finnish individuals with ULs. Here, we were able to identify a significant association between UL and LoF variants in four genes encoding SRCAP complex subunits, *YEATS4*, *ZNHIT1*, *DMAP1*, and *ACTL6A*, and to place mutations in this gene group as a major cause of moderate-penetrance UL predisposition. In our sample collection of Finnish individuals with ULs, examination of tumors from individuals with a germline mutation showed that, similar to the somatic setting, genes encoding SRCAP complex subunits frequently displayed a second hit, inactivating the remaining normal allele. This follows the classical two-hit theory,^27^ where tumors arise from two inactivating mutations targeting both alleles of a tumor suppressor gene.

## Material and methods

### Study subjects

The studied individuals with ULs were from the Finland Myoma Study sample set, which comprised 860 individuals with fresh-frozen ULs and paired normal myometrium. The study has been approved by the Finnish National Supervisory Authority for Welfare and Health (THL/151/5.05.00/2017, THL/723/5.05.00/2018, THL/1300/5.05.00/2019) and the Ethics Committee of the Hospital District of Helsinki and Uusimaa (HUS/2509/2016). ULs and the corresponding normal myometrium tissues were collected from six prospective sample series (M, My, My1000, My5000, My6000, and My8000), which are described in more detail in our previous studies.^7^^,^^14^^,^^28^^,^^29^ The anonymous M-series was collected according to Finnish laws and regulations and approved by the head of the health care unit between 2001 and 2002. A written informed consent was obtained for all samples collected in the subsequent sample series.

**Figure 1:**
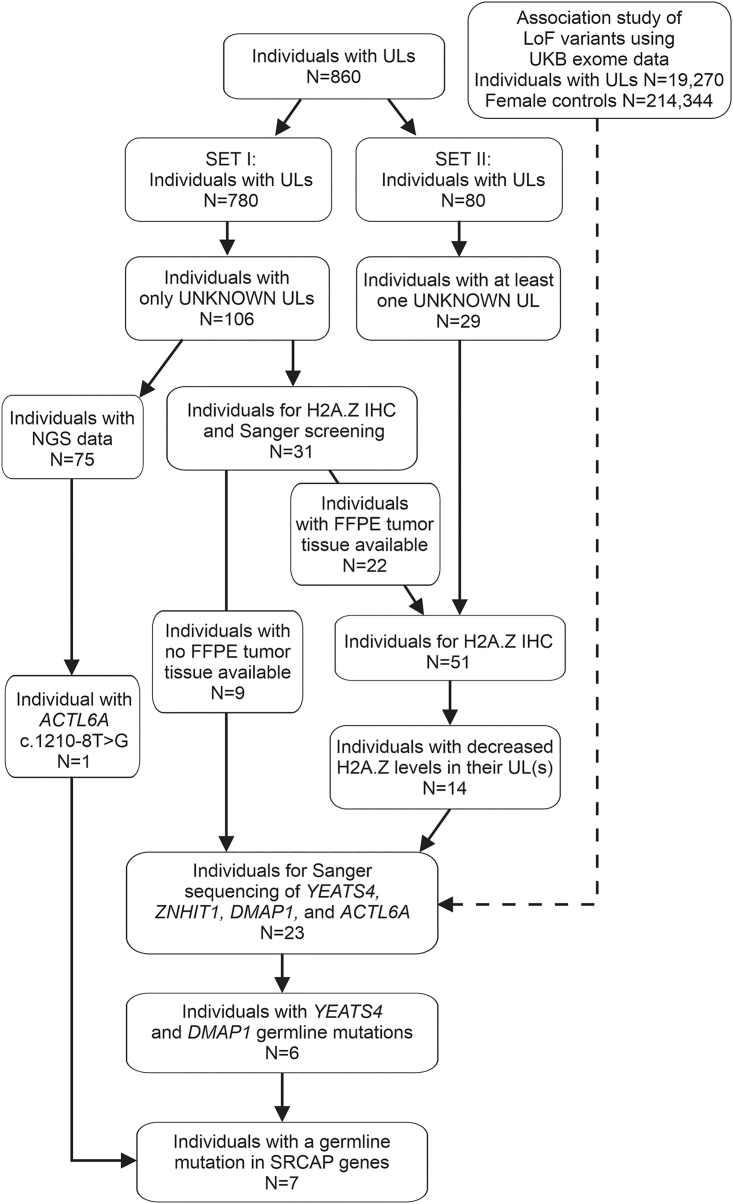
Flowchart of the sample selection process for mutation screening of *YEATS4*, *ZNHIT1*, *DMAP1,* and *ACTL6A* Altogether, 860 individuals with ULs entered the study. SET I contained 106 individuals with no UL driver mutations in their tumors (“UNKNOWN ULs”). RNA-seq or WGS data available from 75 individuals revealed one *ACTL6A* germline mutation. The remaining 31 individuals entered the H2A.Z IHC pre-screening and/or the Sanger sequencing. From SET II, all the UNKNOWN tumors from 29 individuals were selected for the H2A.Z IHC and the subsequent Sanger sequencing. Sanger sequencing revealed two individuals with a *YEATS4* and four with a *DMAP1* germline mutation. Selection criteria for the studied individuals is explained in a more detailed manner in the material and methods section.

The first set of 780 individuals with ULs (SET I) included 726 individuals of our previous study^14^ and 54 individuals of subsequently collected tissue samples. From this set, we excluded all the individuals with somatic *MED12* and *HMGA2* driver alterations or somatic/germline *FH* defects detected in at least one of their ULs. Also, women with somatic driver mutation identified in genes affecting the SRCAP complex were excluded. The somatic/germline status of each identified variant was confirmed by sequencing the corresponding normal myometrium. Thus, 106 individuals were selected for the analysis. Next-generation sequencing (NGS) data (RNA-sequencing [RNA-seq] or whole-genome-sequencing [WGS] data) available from 75 individuals were used to study the the mutational status of the genes encoding SRCAP complex subunits. The remaining 31 individuals entered the screening for mutations affecting the SRCAP complex: for 22 individuals (25 tumors) with UL blocks, available H2A.Z immunostaining was used as a prescreening method, as mutations affecting the SRCAP complex are shown to result in decreased H2A.Z protein levels.^14^ The remaining nine individuals were directly Sanger sequenced (Figure 1). The second set (SET II) contained 80 individuals with ULs. All ULs with no *MED12* or *HMGA2* driver alteration were selected for the screening for mutations affecting the SRCAP complex. First, 34 ULs from 29 individuals entered H2A.Z immunohistochemistry (IHC) prescreening (Figure 1). Subsequently, all the individuals with ULs showing decreased H2A.Z levels entered Sanger sequencing (see details in mutation screening).

### UK Biobank material and LoF associations

The UKB whole-exome-sequencing (WES) material (UKB resource under application number 80756) was processed as follows. We used the improved and unified pipeline (OQFE) variant calls of 450,000 WES individuals (UKB data-field 23149; accessed in March 2022). Variant calls were filtered to have a read depth of at least 10 in at least 90% of individuals (UKB depth filter “90pct10dp”). Variant effect annotations were done with SnpEff (v.5.1; GRCh38.86): variants annotated as stop gained, frameshift, splice acceptor variant, splice donor variant, start lost, or stop lost were included in the analysis and are referred to as LoF variants. Similar to previous work,^30^ we extracted individuals of White European ancestry on the basis of the first two genetic principal components (data-field 22009): after excluding individuals with excessive kinship (data-field 22021), sex chromosome aneuploidy (field 22019), outliers for heterozygosity or missing rate (field 22027), and genetic sex mismatching self-reported sex (fields 31, 22001), a total of 430,728 (95.1%) White European individuals were identified, the majority of which (98.8%) were self-reported “White British” (n = 399,357), “other White background” (n = 14,525), or “White Irish” (n = 11,844). A subset of 233,614 White European females were then inspected to determine their UL phenotype: we used ICD10 (“D25,” field 41270) and ICD9 (“2189,” field 41271) diagnosis codes, OPCS4/3 myomectomy operation codes (fields 41272, 41273), and self-reported ULs (“uterine fibroids”/”myomectomy”) to identify a total of 19,270 individuals with ULs. Individuals who had undergone hysterectomy were identified similarly, resulting in 39,439 additional individuals with this complementary phenotype.

Gene-based association tests were computed with SAIGE-GENE+ (v.1.0.1)^31^^,^^32^ by grouping variants by gene symbol, according to variants’ highest SnpEff annotated impact. Common germline variants (minor allele frequency, MAF > 1%) were excluded from the analysis. All genes with sum(minor allele count, MAC) ≥ 3 were included. We report gene-based associations (SKAT-O test p values) adjusted for age at first assessment center visit (data-field 21003), sequencing batch (different oligo lots in phase 1), and first four genetic principal components (data-field 22009). Since the SKAT-O test does not provide effect size estimates or confidence intervals, we calculated odds ratio (OR) estimates as numbers of individuals stratified by presence of LoF variants and phenotype status. Quantile-quantile plots of SKAT-O test p values were inspected for genomic inflation and to determine an exome-wide significance threshold (Figure S1): the observed p values deviated from the expected null distribution approximately at p < 1e−4, which was chosen as a putative exome-wide significance threshold. The expected number of exome-wide false-discoveries, under the null hypothesis of no effect across all 18,899 genes, was less than two (≈1.89). Given that the focus of our work were the nine genes encoding SRCAP complex subunits, we applied a significance threshold of p < 0.05/9 among the genes encoding SRCAP complex subunits.

A subsequent analysis of age at diagnosis was implemented to further inspect the clinical relevance of LoF variants (two-sided Welch’s t test), including Kaplan-Meier estimator plots and two-sided log-rank test p values (lifelines v.0.26.5). Start of follow-up was set at birth. Follow-up ended at the first record of UL diagnosis, hysterectomy, death, or at censoring date (September 30, 2021), whichever came first. Age information was available for 19,208 UL diagnoses and 51,145 hysterectomies. Age at menarche and menopause were available for 226,859 and 140,232 women, respectively.

### Phenome-wide associations in UK Biobank

We explored the AstraZeneca phewas portal (https://azphewas.com/; accessed on February 3, 2022) for publicly available summary statistics. The portal gives gene-phenotype associations calculated with the exome-sequenced UKB individuals: the publicly available associations cover European ancestry (394,695 individuals), eleven different variant models, and approximately 15,500 binary phenotypes.^33^ Their “ptv” and “ptv5pcnt” models, referred to here as “LoF,” comprise variants annotated as exon loss variant, frameshift variant, start lost, stop gained, stop lost, splice acceptor variant, splice donor variant, gene fusion, bidirectional gene fusion, rare amino acid variant, and transcript ablation. These models include MAF ≤ 0.1% and MAF ≤ 5% variants, respectively. Details of these different variant models and gene-based collapsing analysis (two-sided Fisher’s exact test) can be found in the original publication.^33^ To account for multiple testing across 15,500 phenotypes and nine genes encoding SRCAP complex subunits, quantile-quantile plots were inspected to choose a putative significance threshold of p < 5e−4.

### Moderate-penetrance UL associations in UK Biobank

Summary statistics representing moderate-penetrance UL associations were readily available in the AstraZeneca phewas portal (https://azphewas.com/; accessed on February 3, 2022). We extracted the endpoint “Union#D25#D25 Leiomyoma of uterus,” which was defined as a combination of ICD10 diagnostic code D25 and self-reported ULs.^33^ The resulting phenotype associations were based on White European ancestry and 15,780 individuals with ULs and 197,159 female controls. A gene-based collapsing test (two-sided Fisher’s exact test) and the different categories of variants explored are described in the original publication.^33^ A threshold of OR > 2.0 was used to characterize associations with at least a moderate effect-size.^34^ To account for multiple-testing across ten different variant categories and roughly 19,000 genes, quantile-quantile plots were inspected to determine a significance threshold of p < 1e−4. A synonymous variant model was used as an empirical negative control; we observed two false discoveries at the chosen thresholds (OR > 2.0 and p < 1e−4).

### IHC and histopathology

H2A.Z loading was assessed by IHC utilizing anti-histone H2A.Z antibody (dilution 1:2,500; Abcam ab150402) on formalin-fixed paraffin-embedded (FFPE) sections (5 μm). For detection, the Orion two-component detection system (peroxidase, goat anti-rabbit/mouse IgF HRP [ready-to-use]; WellMed BV, cat. no. T100-HRP) was utilized. As previously reported,^14^ samples were classified on the basis of the immunoreaction intensity into three groups: 0 = negative or weak, 1 = moderate, 2 = strong. Tumor sections harboring mutations in genes encoding SRCAP complex subunits were used as negative controls and *MED12* mutant as positive controls. Histopathological examination was performed by a pathologist. Immunostained tissue sections were imaged with Qupath.^35^ If any of the tumor sections of the individual showed reduced H2A.Z immunoreactivity, the individual was selected for capillary sequencing of *YEATS4*, *ZNHIT1*, *DMAP1*, and *ACTL6A* (see details in mutation screening).

### Mutation screening

Mutation screening from WGS and RNA-seq data was done as previously described.^7^^,^^14^^,^^36^^,^^37^

For capillary sequencing, genomic DNA was extracted from fresh frozen UL and myometrium samples with QIAamp FAST DNA Tissue Kit and from FFPE samples with GeneRead DNA FFPE Kit (Qiagen). RNA was isolated by TRIzol Reagent (Invitrogen), RNase-free Dnase (Qiagen), and Rneasy MiniElute clean-up kit (Qiagen). RNA was converted to cDNA according to standard procedures. The germline mutation status of genes encoding SRCAP complex subunits was confirmed by Sanger sequencing of both ULs and corresponding normal myometrium sample. PCR amplifications and Sanger sequencing was performed as previously described.^7^ Primers were designed with the Primer3-program (https://primer3.ut.ee/)^38^ (Table S1). All coding exons of *YEATS4*, *ZNHIT1*, *DMAP1*, and *ACTL6A* were sequenced to detect potential germline mutations.

### Variant effect prediction and gnomAD

The gnomAD^39^ v.2.1.1 database (https://gnomad.broadinstitute.org/) was used as a resource for population frequencies of the germline mutations. To predict the pathogenicity of the variants, PolyPhen-2^40^ v.2.2.3r406 (http://genetics.bwh.harvard.edu/pph2/), SIFT^41^^,^^42^ v5.1.1 (http://sift-dna.org), and SpliceAI^43^ (https://spliceailookup.broadinstitute.org/, accessed on October 17, 2022) were used.

### Nanopore long-read sequencing

Long-read sequencing was performed as previously described.^14^ Briefly, the libraries were prepared with a Ligation Sequencing Kit (Oxford Nanopore Technologies), following the manufacturer’s Genomic DNA by Ligation protocol. The PromethION platform was used with MinKnow-Live-Basecalling (v.3.4.6) for sequencing and base calling. Reads were aligned against the reference genome GRCh38 with minimap2 (v.2.16)^44^ and phased to haplotypes with Longshot (v.0.4.0).^45^ NanoStat (v.1.1.2) and NanoPlot (v.1.20.0)^46^ were used to evaluate the quality of the data. Methylation statuses of the CpG sites were called by F5C.^47^ Integrative Genomics Viewer (IGV) was used for the visualization of the *YEATS4* mutation site. The average methylation values for 1,000 bp upstream from the *YEATS4* transcription start site were calculated specifically for both alleles and visualized with R packages called datatable (v.1.13.6) and tidyverse (v.1.3.0).

## Results

### LoF of genes encoding SRCAP complex subunits is a major contributor to UL predisposition

An outline of our study is given in Figure 1. We analyzed germline LoF variants across 18,899 genes and 233,614 exome-sequenced White European women for association to UL. A gene-based analysis of 19,270 individuals with ULs and 214,344 female controls revealed significant associations to *YEATS4* (OR = 5.8; p = 3.1e−11; SKAT-O test) and *ZNHIT1* (OR = 3.3; p = 2.6e−8), appearing as the top two associations out of all genes inspected (Table 1). Two additional genes, *DMAP1* (OR = 2.2; p = 5.0e−5) and *ACTL6A* (OR = 7.0; p = 1.6e−3), out of the nine genes encoding SRCAP complex subunits were also significant (p < 0.05/9) and ranked seventh and 46th out of 18,899 genes, respectively. Mutations in the remaining five genes encoding SRCAP complex subunits did not show association to UL in this analysis (Table 1). Outside the genes encoding SRCAP complex subunits, significant UL associations (at p < 1e−4) were identified for *CHEK2* (OR = 1.5; p = 1.1e−6), *ATM* (OR = 1.7; p = 1.4e−5), *PTTG1* (OR = 7.7; p = 2.5e−5), *BEND3* (OR = 8.9; p = 3.9e−5), *ANGPT4* (OR = 3.1; p = 7.3e−5), *EPN2* (OR = 2.1; p = 8.8e−5), and *DCLRE1A* (OR = 1.3; p = 9.7e−5).

**Table 1:** Germline loss-of-function variant associations to uterine leiomyoma

**Gene**	**Number of variants**^a^	**Ultra-rare variants**^b^	**MAC**^c^**(ULs)**	**MAC**^c^**(controls)**	**Rank**	**OR**	**p**
*YEATS4*^d^	2	13	30	58	1	5.8	3.1e−11
*ZNHIT1*^d^	3	9	26	89	2	3.3	2.6e−8
*CHEK2*	9	40	172	1,270	3	1.5	1.1e−6
*ATM*	13	195	91	604	4	1.7	1.4e−5
*PTTG1*	0	10	9	13	5	7.7	2.5e−5
*BEND3*	0	11	8	10	6	8.9	3.9e−5
*DMAP1*^d^	4	22	30	153	7	2.2	5.0e−5
*ANGPT4*	1	20	13	47	8	3.1	7.3e−5
*EPN2*	2	29	41	220	9	2.1	8.8e−5
*DCLRE1A*	3	55	247	2,118	10	1.3	9.7e−5
*ACTL6A*^d^	0	8	5	8	46	7.0	1.6e−3
*SRCAP*	1	13	4	23	6,856	1.9	0.4
*VPS72*	1	19	5	74	12,708	0.8	0.7
*ACTR6*	1	26	5	65	15,418	0.9	0.8
*RUVBL2*	0	6	1	11	17,411	1.0	1.0
*RUVBL1*	0	2	0	2	N/A^e^	N/A^e^	N/A^e^

Summary statistics for 19,270 individuals with uterine leiomyomas (ULs) and 214,344 female controls. Genes that passed exome-wide significance at p < 1e−4 are shown, including a summary of all nine genes encoding SRCAP complex subunits. The p values are from gene-based SKAT-O tests. OR, odds ratios; rank, rank among all 18,899 genes.

a Number of variants with minor allele frequency (MAF) ≤ 1%, excluding ultra-rare variants.

b Number of ultra-rare variants with minor allele count (MAC) ≤ 10.

c Minor allele count (MAC) among individuals with ULs and female controls.

d Genes encoding SRCAP complex subunits that passed p < 0.05/9.

e N/A: no statistics were computed for minor allele count < 3 genes.

We then complemented our association analysis with individuals that had undergone hysterectomy, representing a proxy for any potentially missing UL diagnoses and a more severe phenotype for our analysis. Of note, 12,123 of the 19,270 individuals with ULs (63%) had undergone hysterectomy. The combined phenotype gave us altogether 58,709 individuals with ULs or hysterectomy and 174,905 female controls for the analysis. The four genes encoding SRCAP complex subunits showed a striking association also to this alternative phenotype: *YEATS4* (OR = 5.0; p = 5.7e−13), *ZNHIT1* (OR = 2.6; p = 3.0e−11), *DMAP1* (OR = 1.8; p = 1.2e−5), and *ACTL6A* (OR = 6.7; p = 4.8e−4), all within top 21 strongest associations out of the 18,899 genes tested (Figure S1). No associations were identified for the remaining five genes encoding SRCAP complex subunits. Other genes with a notable hysterectomy risk (at p < 1e−9) were *MSH6* (OR = 3.3; p = 1.9e−20) and *BRCA1* (OR = 1.9; p = 9.5e−10), most likely explained by susceptibility to endometrial and ovarian cancers, respectively^48^^,^^49^ (Table S2). Additional significant associations were identified for *CHEK2* (OR = 1.4; p = 6.4e−8), *MSH2* (OR = 3.1; p = 9.2e−6), *NAE1* (OR = 2.6; p = 1.2e−5), *FH* (OR = 3.6; p = 1.5e−5), *MLH1* (OR = 1.8; p = 3.4e−5), *GREB1* (OR = 2.1, p = 5.0e−5), and *ACAT2* (OR = 0.97; p = 7.0e−5).

### Germline LoF gene mutations that affect the SRCAP complex associate with younger age at UL diagnosis

**Figure 2:**
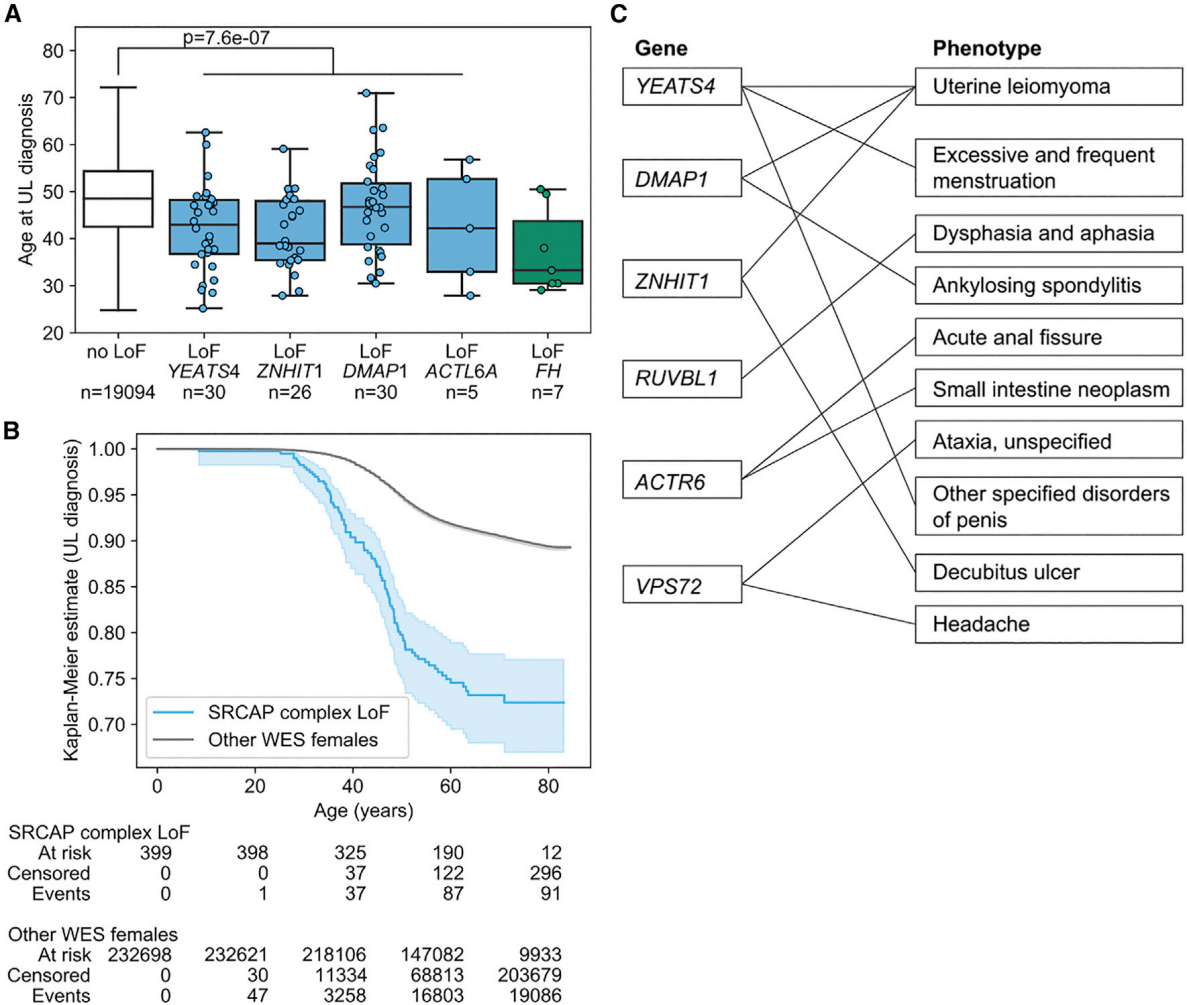
Analysis of age at UL diagnosis and phenome-wide associations (A and B) *YEATS4*, *ZNHIT1*, *DMAP1*, and *ACTL6A* loss-of-function (LoF) variants contribute to younger age at UL diagnosis (p = 7.6e−7; two-sided Welch’s t test). (A) Age at UL diagnosis stratified by the gene with an LoF variant. Boxplots show the median and the first and third quartiles of the data; whiskers extend up to 1.5 interquartile range and dots show the individual observations. *FH* is shown as a reference. No LoF, individuals without LoF variants among the genes highlighted. (B) Kaplan-Meier estimates (p = 8.7e−37; two-sided log-rank test) and at risk, censored, and event counts for the five time points on the x axis. Individuals with *FH* LoF variants were excluded from the Kaplan-Meier estimates. The 95% confidence interval is shown as a translucent band (lifelines plot; see material and methods). (C) Phenome-wide associations for LoF mutations affecting the SRCAP complex. The nine genes encoding SRCAP complex subunits were tested for LoF variant associations across 15,500 phenotypes. Gene-based collapsing tests that passed p < 0.0005 (two-sided Fisher’s exact test) are shown connected by a line between the respective gene and phenotype. Detailed summary statistics for each gene-phenotype pair are given in Table S3.

The four genes encoding SRCAP complex subunits, *YEATS4*, *ZNHIT1, DMAP1*, and *ACTL6A*, were further examined for LoF variants’ impact on age at diagnosis, menarche, and menopause. Age at diagnosis information was available for 19,208 individuals with ULs and 51,145 individuals that had undergone hysterectomy. Individuals with an LoF variant in *YEATS4*, *ZNHIT1*, *DMAP1*, or *ACTL6A* displayed a significantly younger age at UL diagnosis (mean 43.2 years) than expected (mean 48.8 years; p = 7.6e−7; Welch’s t test; Figure 2). Similar difference was observed when hysterectomies were included in the analysis: individuals with an LoF variant in these four genes displayed a significantly younger age (mean 43.1 years) at UL diagnosis or at hysterectomy, whichever came first, than expected (mean 46.9 years; p = 6.9e−8; Welch’s t test; Figure S1). LoF variants in these four genes had no significant effect on age at menarche or menopause (Figure S2).

### Phenome-wide analysis of LoF mutations affecting the SRCAP complex

We explored a recently published repository of phenome-wide associations in order to understand whether LoF mutations affecting the SRCAP complex contribute to any other phenotypes besides UL. The repository, referred to as the AstraZeneca portal, gives gene-phenotype associations across 394,695 exome-sequenced individuals of European ancestry and 15,500 binary phenotypes.^33^ They provide a gene-based burden test of LoF variants for all gene-phenotype pairs; note that these 15,500 binary phenotypes were computationally defined^33^ and may contain substantial redundancy between the endpoints. We inspected the summary statistics of all nine genes encoding SRCAP complex subunits: a total of 27 associations passed a putative significance threshold (at p < 0.0005; two-sided Fisher’s exact test), nine of which were alternative, computationally generated definitions of the UL endpoint (Table S3). A summary of the resulting associations is given in Figure 2C. Aside from the UL endpoint, the next two strongest associations were *YEATS4* association to excessive and frequent menstruation (p = 8.1e−7; two-sided Fisher’s exact test), most likely explained by comorbidity with UL, and *RUVBL1* association to dysphasia and aphasia (p = 1.7e−6). The latter association had extremely low numbers—altogether only two individuals with dysphasia/aphasia and *RUVBL1* LoF variants identified—preventing meaningful interpretation and further explanatory analyses. The remaining phenome-wide associations of genes encoding SRCAP complex subunits (Table S3) were similarly difficult to interpret as a result of very low numbers of affected individuals (n ≤ 8 individuals with LoF mutations) and orders of magnitude weaker significance levels (p > 1e−5). Among all the 15,500 phenotypes examined, the UL phenotype emerged as the only endpoint with a robust association to LoF variants in genes encoding SRCAP complex subunits.

### Moderate-penetrance predisposition to UL

We then focused on moderate-to-high risk UL predisposition, taking into account a more comprehensive set of coding-region alterations than just LoF variants. To this end, exhaustive gene-level associations were available from the AstraZeneca portal, for an endpoint comprising 15,780 individuals with ULs and 197,159 female controls.^33^ We inspected all available summary statistics across ten different variant categories and 19,000 genes for associations to UL. In brief, the ten non-synonymous variant categories included nine dominant and one recessive model.^33^ An additional synonymous variant model was included as an empirical control of false-discoveries. We chose a commonly accepted threshold of OR > 2.0 to characterize associations with a moderate or higher effect size.^34^ A total of eleven associations, arising from eight genes, passed a significance level of p < 1e−4 (gene-based collapsing test, Figure S3). Three of these moderate-penetrance UL associations were attributed to LoF variants at *YEATS4* (OR = 5.0; p = 1.4e−8), *DMAP1* (OR = 3.7; p = 1.6e−6), and *ZNHIT1* (OR = 3.1; p = 2.6e−5). Rare non-synonymous, *in silico* predicted damaging variants (MAF ≤ 0.1%) displayed moderate-penetrance associations to UL at *YEATS4* (OR = 3.1; p = 1.5e−5), *SRPK2* (OR = 2.1; p = 2.8e−5), *MMP11* (OR = 2.2; p = 5.9e−5), and *ZNHIT1* (OR = 2.2; p = 9.9e−5). Ultra-rare variants (MAF ≤ 0.005%) had a moderate-penetrance association at *TMED7* (OR = 6.0; p = 2.1e−5; n = 11 individuals with ULs and a qualifying variant), and the recessive model displayed two associations at *ZNF697* (OR = 10.9; p = 4.6e−5) and *DNASE2* (OR = 6.3; p = 9.2e−5), both with low numbers of individuals with ULs (n = 7 and 9 with a qualifying variant, respectively; Figure S3).

### H2A.Z immunohistochemistry prescreening of mutations affecting the SRCAP complex

From the 106 individuals with ULs selected for the analysis (SET I), NGS data was available from ULs of 75 women (Figure 1). Focusing on mutations affecting genes encoding SRCAP complex subunits, we identified an *ACTL6A* variant in one UL (My6105m1),^14^ and the tissue samples of the individual entered additional validations as described below. Variants in the genes encoding SRCAP complex subunits were not detected in any other individual via NGS data (Figure 1). The remaining 31 individuals without NGS data entered additional studies. From SET II, all tumors with no *MED12* or *HMGA2* UL driver alterations, 34 ULs from 29 individuals were selected for further studies.

As we have recently shown, somatic mutations in genes encoding SRCAP complex subunits result in defective deposition of the histone variant H2A.Z, which can be seen as a weak/absent H2A.Z IHC staining in ULs.^14^ Therefore, H2A.Z IHC was used as a prescreening method to detect mutations affecting the SRCAP complex. From the 60 individuals (SET I and II) selected for additional studies, FFPE blocks were available from 51 individuals (Figure 1). All 59 respective tumors entered the H2A.Z immunostaining. Altogether, 16 ULs from 14 individuals displayed decreased H2A.Z immunostaining (staining classified as 0 or 1) (Table S4). No reduction of H2A.Z staining was observed in the corresponding normal myometrium of these individuals. These 14 individuals together with nine individuals from SET I with no available FFPE blocks were further analyzed by *YEATS4*, *ZNHIT1*, *DMAP1*, and *ACTL6A* Sanger sequencing.

### Identification and validation of germline mutations affecting the SRCAP complex

In our previous study, we identified by RNA-seq two *ACTL6A* mutations, c.85_86delinsTT (GenBank: NM_004301.5) (p.Gly29Phe) and intronic point mutation c.1210−8T>G (GenBank: NM_004301.5) (p.Gly404Phefs∗16) in the UL of individual My6105.^14^ The somatic nature of c.85_86delinsTT (exon 2) was verified, but the origin of c.1210−8T>G variant 8 bp upstream of exon 14 was not further validated in that study. Subsequent analysis by SpliceAI predicted this mutation to cause an acceptor gain with a delta score of 0.94. DNA and cDNA sequencing revealed that c.1210−8T>G is a germline alteration resulting in a splicing defect generating a new intron-exon boundary −7 bp from the canonical splice site (r.1209_1210insTTTACAG) (Figure S4). The minor allele was not present in the gnomAD germline variant database. Sequencing of the individual’s three UL samples revealed a somatic *ACTL6A* mutation in all tumors, implicating a classic “two-hit” inactivation of the gene.^27^ c.85_86delinsTT (p.Gly29Phe) was found in UL My6105m1, My6105m4 harbored a frameshift insertion c.578_579insTTCATAGGCATTGT (GenBank: NM_004301.5) (p.Lys194Serfs^∗^2), and Nanopore long-read sequencing showed a duplication of exon 12 in UL My6105m5 (Table 2). This individual was 33 years old at the time of diagnosis, and hysterectomy was performed at the age of 38 because of heavy menstrual bleeding and pelvic pressure. The weight of the uterus was 682 g and it contained five large (diameter 15–60 mm) and multiple smaller ULs (Table 3). In addition, this individual had undergone surgery for lipoma. Family history information for UL was not available.

**Table 2:** Genetic information of the individuals with uterine leiomyomas and a germline mutation in one of the genes encoding SRCAP complex subunits

**Individual ID**	**Germline mutation**^a^	**AF**^b^**in gnomAD v.2.1.1 (Finnish)**	**AF**^b^**in gnomAD v.2.1.1 (total)**	**Myoma ID**	**Second hit**^a^	**Mutation in another driver gene**^a^
**My6105**	*ACTL6A* c.1210−8T>G (p.Gly404Phefs∗16)	0	0	My6105m1	c.85_86delinsTT (p.Gly29Phe)	–
				My6105m4	c.578_579insTTCA TAGGCATTGT (p.Lys194Serfs^∗^2)	–
				My6105m5	duplication of exon 12^c^	–
**My6564**	*YEATS4* c.74T>C (p.Ile25Thr)	0	0.00001998	My6564m1	hypermethylation	–
**My6606**	*YEATS4* c.74T>C (p.Ile25Thr)	0	0.00001998	My6606m1	N/A^d^^,^^f^	–
				My6606m2	N/A^d^^,^^f^	–
				My6606m3	N/A^d^^,^^f^	–
				My6606m4	hypermethylation	–
				My6606m5	N/A^d^^,^^f^	–
				My6606m6	hypermethylation	–
**My6589**	*DMAP1* c.907−5C>G	0.003988	0.0007274	My6589m1	N/D^e^^,^^f^	–
**My6621**	*DMAP1* c.1247C>T p.(Pro416Leu)^g^	0.001397	0.0004105	My6621m1	N/D^e^^,^^f^	–
**My6638**	*DMAP1* c.1158T>G (p.Tyr386^∗^)	0	0	My6638m1	c.79A>T (p.Lys27^∗^); c.85G>T (p.Asp29Tyr)	–
				My6638m2	c.511T>G (p.Phe171Val)	–
				My6638m3	c.199delG (p.Asp67Metfs^∗^17)	–
				My6638m4	N/D^e^^,^^f^	*MED12* c.83_99+1del18
**My6660**	*DMAP1* c.409G>C (p.Val137Leu)^g^	0.005693	0.0008837	My6660m1	N/D^e^^,^^f^	–

a Variants and mutations given according to the following GenBank reference sequences, GenBank: NM_004301.5 (*ACTL6A*), NM_006530.4 (*YEATS4*), NM_019100.5 (*DMAP1*), NM_005120.3 (*MED12*).

b AF, allele frequency.

c Duplicated region chr3: 179,583,28 –179,583,524 (GRCh38).

d N/A, information not available.

e N/D, not detected.

f Methylation data not available.

g Pathogenicity uncertain, predicted tolerated by SIFT and benign by PolyPhen-2.

**Table 3:** Clinical and histopathological information about individuals with uterine leiomyomas and a germline mutation in one of the genes encoding SRCAP complex subunits

**Individual ID**	**My6105**	**My6564**	**My6606**	**My6589**	**My6621**^a^	**My6638**	**My6660**^a^
**Clinical information**							

**Number and size of ULs**^b^^**,**^^c^	five (∅ 15 –60 mm), ∼5 smaller	one (∅ 110 mm)	six (∅ 30 –90 mm), ∼50 smaller	one (∅ 97 mm)	two (∅ 32 and 7 mm)	four (∅ 15 –60 mm)^d^	one (∅ 32 mm)
**Weight of the uterus**	682 g	612 g	1490 g	387 g	168 g	145 g	160 g
**Tumor location (submucous/intramural/subserous)**	intramural, partly subserous	intramural	intramural	N/A^e^	submucous and intramural	one intramural partly protruding to submucosa, two intramural, one subserous	intramural partly protruding to submucosa
**Age at diagnosis**	33	42	42	28	30	47	38
**Age at hysterectomy**	38	46	44	41	38	52	40
**Heavy menstrual bleeding**	yes	Yes	yes	yes	yes	yes	yes
**Pelvic pressure/pain**	yes	No	yes	yes	yes	yes	no
**Anemia**	N/A^e^	No	yes	no	yes	yes	yes
**Other tumors**	lipoma	No	no	no	no	no	breast cancer

**Family history**							

**Family history of ULs**^b^	N/A^e^	mother, grandmother and 3 aunts	N/A^e^	aunt	N/A^e^	mother	mother
**Family history of hysterectomies**	N/A^e^	mother, grandmother and 3 aunts	mother and mother’s mother	aunt	mother	mother	no

**Histopathology**							

**Cellular**	yes	No	yes	yes	yes	yes (m1-m2), no (m3-m4)	no
**Nuclear atypia**	no	No	mild in some tumors	mild	no	no	no
**Mitotic count < 1/10 HPF**^f^	yes	Yes	yes	yes	yes	yes	yes
**Atypical mitosis**	no	No	no	no	no	no	no
**Ischemic degeneration**	mild	No	mild	mild	no	yes (m1, m3-m4), no (m2)	strong
**Tumor cell necrosis**	no	No	no	no	no	no	no
**Aberrant vasculature**	no	No	no	no	no	no	no
**Inflammation**	no	N/A^e^	no	mild	no	no	no

a Pathogenicity of the germline mutation uncertain, predicted tolerated by SIFT and benign by PolyPhen-2.

b UL, uterine leiomyoma.

c ∅, diameter.

d The largest tumor was ∼60mm prior to presurgical Esmya treatment.

e N/A, information not available.

f HPF, high power fields.

**Figure 3:**
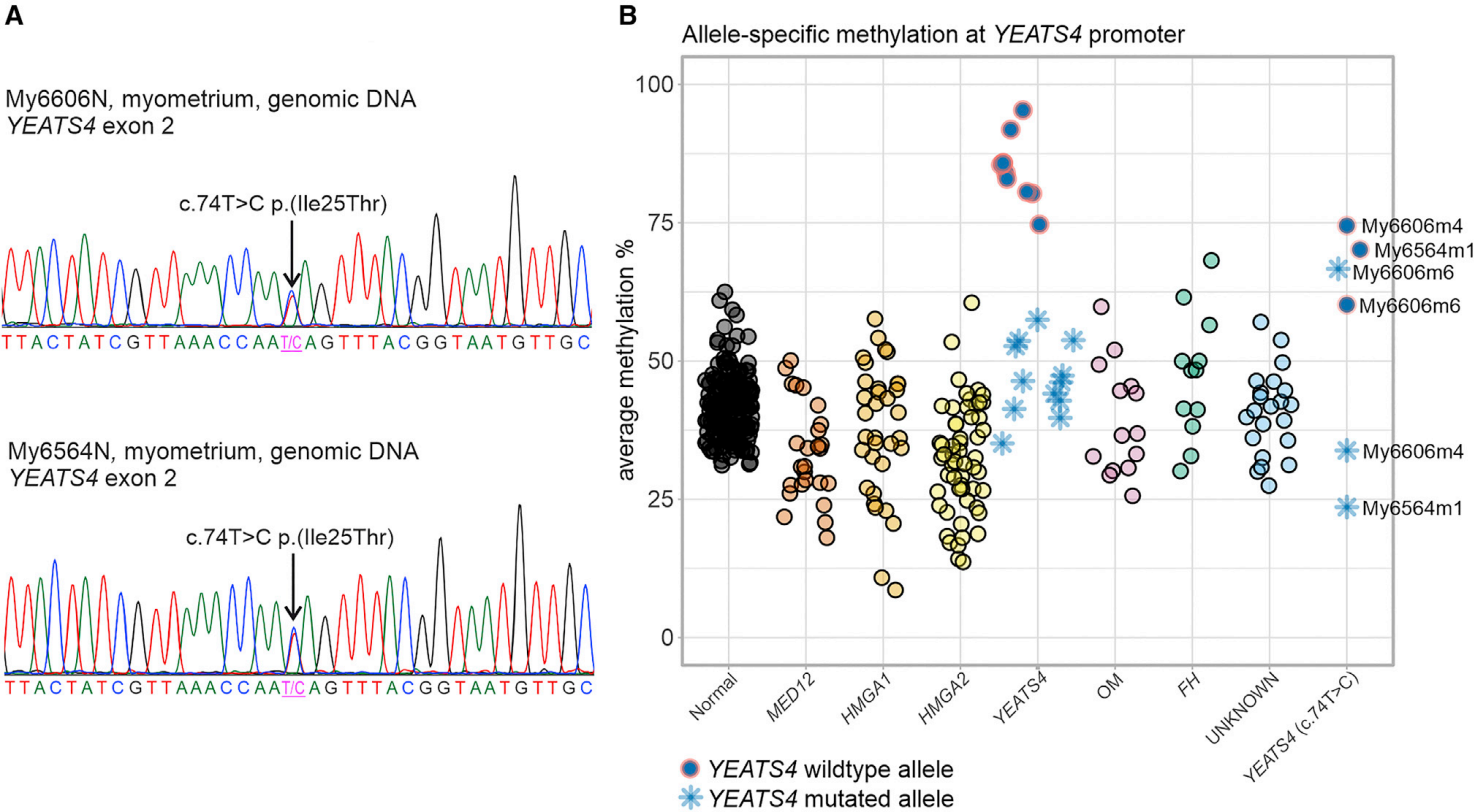
An inherited mutation of *YEATS4* and promoter hypermethylation of the other allele resulted in bi-allelic inactivation of *YEATS4* in the ULs of two individuals (A) Individuals My6606 and My6564 had a germline missense mutation c.74T>C (p.Ile25Thr) in *YEATS4* exon 2. This mutation was validated to be inherited by Sanger sequencing. (B) Methylation analysis of the region of 1,000 bp upstream from the *YEATS4* transcription start site was done to evaluate promoter methylation. Samples are grouped into normal myometrium samples, six UL subgroups with somatic alterations in known driver genes (*MED12*, *HMGA1*, *HMGA2*, *YEATS4*, other genes encoding SRCAP complex subunits [OM], and *FH*), and ULs without a known driver gene mutation (“UNKNOWN”). The last group (*YEATS4* c.74T>C) consists of three ULs from the individuals My6564 and My6606 with the *YEATS4* c.74T>C germline mutation.

As described above, altogether 23 individuals were selected for Sanger sequencing of *YEATS4*, *ZNHIT1*, *DMAP1*, and *ACTL6A* (Figure 1). Two individuals, My6564 and My6606, harbored a heterozygous germline *YEATS4* c.74T>C (GenBank: NM_006530.4) (p.Ile25Thr) mutation in exon 2 (Figure 3). In the gnomAD database, MAF was 2.0e−5. SIFT and PolyPhen-2 programs predicted that the impact of p.Ile25Thr amino acid substitution is damaging or probably damaging, respectively. We have shown that somatic mutations of *YEATS4* are accompanied with hypermethylation of the wild-type (WT) allele resulting in bi-allelic silencing of the gene.^14^ We used long-read Nanopore sequencing to measure the methylation signal in three tumors of individuals with the *YEATS4* germline mutation: My6564m1, My6606m4, and My6606m6. The Nanopore results implied inactivation of *YEATS4* in all studied leiomyomas. Both in My6564m1 and My6606m4, the WT allele of *YEATS4* was hypermethylated. In UL My6606m6, both alleles of *YEATS4* were hypermethylated (Figure 3). Both individuals with the *YEATS4* c.74T>C mutation were diagnosed with ULs at the age of 42 and had suffered from heavy menstrual bleeding. Individual My6564 with a single UL (diameter 110 mm) had a strong family history for the disease. Her mother, grandmother, and three aunts had all been diagnosed with ULs and undergone hysterectomy. Individual My6606 had six large (diameter 30–90 mm) and ∼50 smaller ULs, increasing the weight of the uterus to 1,490 g (Table 3). Her mother and mother’s mother had both undergone hysterectomy.

Sanger sequencing revealed heterozygous germline mutations of *DMAP1* in four individuals. Individual My6589 harbored *DMAP1* c.907−5C>G (GenBank: NM_019100.5) with a gnomAD MAF of 7.3e−4, located close to the splice acceptor site next to the 5′ end of exon 7. SpliceAI predicted a possible splice acceptor loss for this mutation with a low delta score of 0.03. cDNA sequencing revealed intron retention of intron 7 at the transcript level (Figure S4). Individual My6638 displayed a nonsense variant *DMAP1* c.1158T>G (GenBank: NM_019100.5) (p.Tyr386^∗^) that was not found in gnomAD. Sequencing revealed somatic second hits in three out of four UL samples available (Table 2). The only UL without a second hit contained a somatic *MED12* driver mutation and may thus have been incidental. Two individuals harbored *DMAP1* missense variants; My6621 had c.1247C>T (GenBank: NM_019100.5) (p.Pro416Leu) mutation with a gnomAD MAF of 4.1e−4 and My6660 had c.409G>C (GenBank: NM_019100.5) (p.Val137Leu) with a gnomAD MAF of 8.8e−4. These two missense variants were predicted to be tolerated by SIFT and benign by PolyPhen-2. The number of ULs varied from one to four in individuals with a *DMAP1* germline variant, and the size of the largest tumor ranged from 32 mm to 97 mm. All four of these individuals with *DMAP1* germline variants had a family history of ULs and/or hysterectomy (Table 3). All four suffered from heavy menstrual bleeding and three of them also from pelvic pressure. Individual My6660 had also had breast cancer, and individuals My6589 and My6621 had a family history of breast cancer on the maternal side.

**Figure 4:**
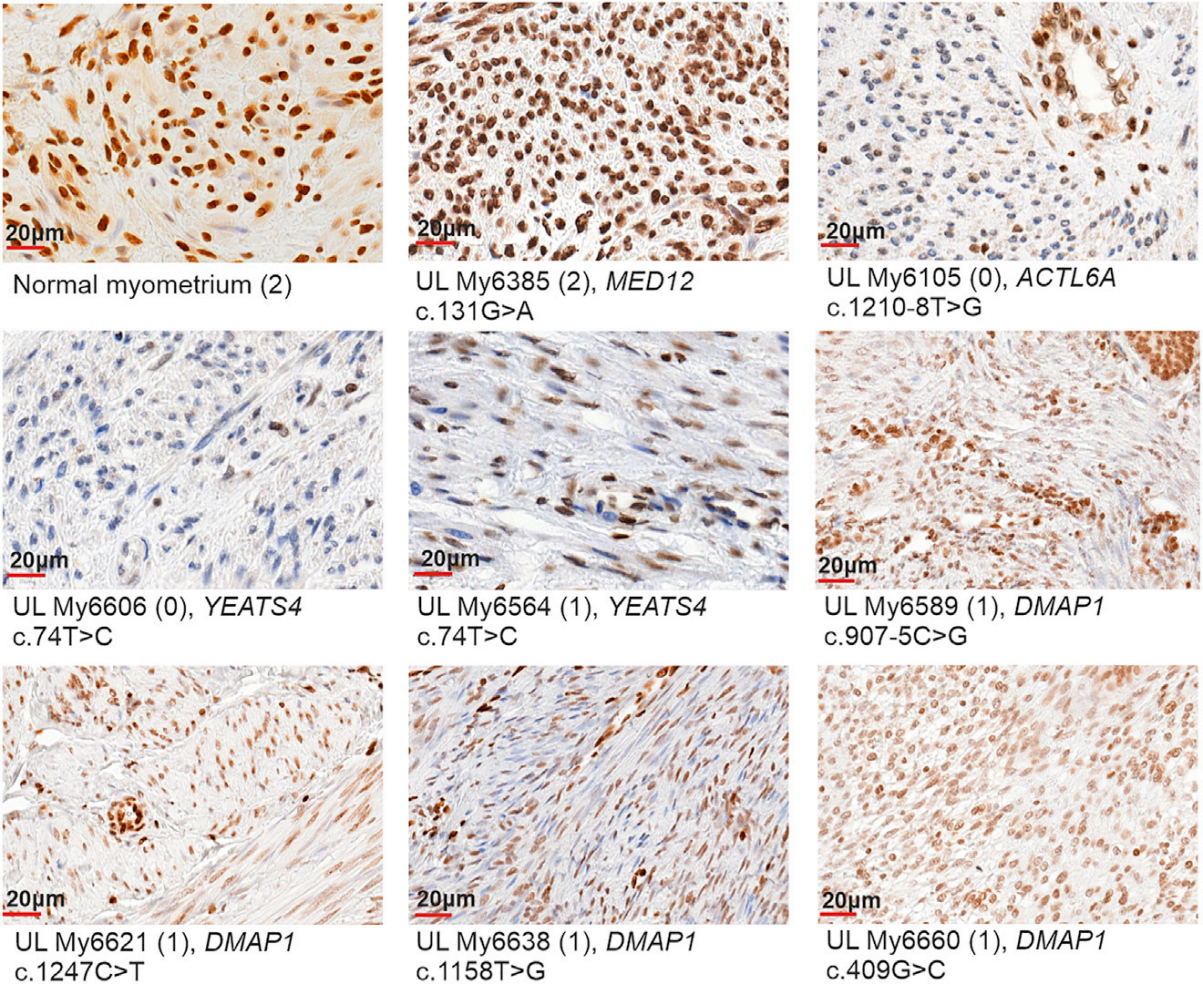
Representative immunostaining of H2A.Z in normal myometrium, *MED12*-mutated UL, and ULs with germline mutations in *ACTL6A*, *DMAP1*, and *YEATS4* Normal myometrium and UL with somatic *MED12* c.131G>A (GenBank: NM_005120.3) (p.Gly44Asp) mutation were used as positive controls. Neoplastic cells of *ACTL6A*-, *YEATS4*-, and *DMAP1*-mutated ULs show negative or very weak (My6105, My6606) to moderate (My6564, My6589 My6621, My6638, My6660) H2A.Z staining but have preserved staining in capillary wall and isolated positive lymphocytes. The intensity of immunoreaction in parentheses: 0 = negative/weak; 1 = moderate; 2 = strong. The scores for all stained sections are presented in Table S5. The scale bars show 20 μm.

To study whether identified germline mutations affecting the SRCAP complex affect H2A.Z protein levels, we performed H2A.Z immunostaining. ULs from all seven individuals with a germline mutation in *ACTL6A*, *YEATS4*, or *DMAP1* showed absent/weak (0) or moderate (1) H2A.Z nuclear immunostaining (Figure 4, Table S5).

## Discussion

The great majority of ULs are caused by somatic alteration in the established driver genes. The genetic driver underlying UL genesis remains unknown for about 10% of tumors.^14^ A high degree of heritability in the development of ULs has been demonstrated by twin studies, familial aggregation, and differences in the UL population incidence.^17^^,^^18^^,^^22^^,^^23^ ULs can be associated with tumor predisposition syndromes, including HLRCC, Cowden syndrome, and Schwannomatosis, caused by germline mutations in *FH*, *PTEN*, and *SMARCB1*, respectively.^10^^,^^25^^,^^26^ These genes, however, explain only a minor proportion of the estimated heritability of ULs. We recently reported that somatic mutations affecting genes encoding SRCAP complex subunits play a role in the genesis of some ULs, characterized by deficient loading of variant histone H2A.Z.^14^ We also performed a focused exploration of preliminary exome data from 25,506 women and found that hereditary LoF mutations in two of the genes encoding SRCAP complex subunits, *ZNHIT1* and *YEATS4*, predispose to ULs.^14^

In this study, we performed a comprehensive evaluation of all protein-coding genes by using an extended exome-sequencing cohort of 233,614 White European women and identified LoF variants in four genes encoding SRCAP complex subunits, *YEATS4*, *ZNHIT1*, *DMAP1*, and *ACTL6A*, as a major contributor to UL predisposition. Large-scale sequencing cohorts, such as the UKB, are required to identify germline determinants with an effect size between traditional GWASs and family studies. In our analysis of 19,270 individuals with ULs and 214,344 female controls, LoF of *YEATS4* and *ZNHIT1*, which are genes encoding SRCAP complex subunits, ranked as the two strongest UL associations overall, followed closely by *DMAP1* and *ACTL6A* associations. All four of these associations had effect size estimates (OR 2.2–7.0) surpassing the next two best associations, *CHEK2* (OR = 1.5) and *ATM* (OR = 1.7). Similarly striking is that LoF of genes encoding SRCAP complex subunits was linked exclusively to ULs and, based on phenome-wide results across 15,500 phenotypes, had no clear associations to other neoplasia or disease. This was compatible with our previous study^14^ reporting that somatic mutations affecting the SRCAP complex are specific to UL and are not listed in the Cancer Gene Census (COSMIC v.91). Clinical relevance of our findings was further highlighted by younger age at UL diagnosis and by complementary association to hysterectomy. Finally, we provided a comprehensive characterization of UL predisposition by extending our analysis toward other coding-region alterations than just LoF variants. This included rare non-synonymous, predicted-damaging variants, and underpinned the outstanding role of mutations affecting genes encoding SRCAP complex subunits in moderate-to-high UL risk. These cohort-based findings led us to investigate the presence and role of germline variants affecting the SRCAP complex in our sample collection of Finnish individuals with ULs.

We found seven individuals with a germline mutation in a gene encoding SRCAP complex subunits among our sample collection of 860 individuals with ULs (minimum prevalence of 0.81% [7/860]). The identified predisposing mutations affecting genes encoding SRCAP complex subunits are comparable with the somatic mutations seen in ULs without a germline alteration in genes encoding SRCAP complex subunits.^14^ Individuals with a predisposing mutation affecting the SRCAP complex may of course also develop incidental ULs caused by another somatic UL driver. Indeed, in the current study, individual My6638 from SET II with three ULs driven by bi-allelic *DMAP1* mutations also had one *MED12* mutated UL (Table 2). Thus, we might have missed individuals with predisposing mutations affecting the SRCAP complex as a result of the selection criteria used in SET I; individuals with common somatic driver alterations in at least one of their ULs were excluded from the study. However, it has been noted that tumors with mutations in *MED12* and genes encoding SRCAP complex subunits aggregate to a subset of individuals instead of being randomly distributed among individuals.^7^^,^^14^^,^^29^ This finding together with mutual exclusivity of UL drivers is an indicator that individuals with the somatic UL driver alterations in their tumors are not the best candidates for UL predisposition screening. Thus, we do believe that this criterion was useful in enriching tumors from the individuals likely to harbor predisposing variants in genes encoding SRCAP complex subunits.

The first detected predisposing mutation was a heterozygous *ACTL6A* splice defect, and tumors of the individual displayed bi-allelic inactivation of the gene by somatic second hit events (Table 2), a feature characteristic for tumor suppressor genes.^27^ Another identified germline defect was a heterozygous missense mutation in *YEATS4* that was detected in two individuals, and the other *YEATS4* allele was silenced in ULs by promoter hypermethylation (Table 2). *DMAP1* germline mutations were detected in four individuals: one splice-site, one nonsense and two missense mutations. The individual with the nonsense mutation had somatic second hits of *DMAP1* in three out of four ULs. The only tumor without a somatic second hit was *MED12* mutation positive (Table 2). This finding supports our previous observation that UL driver alterations are typically mutually exclusive.^14^

In individual My6589 with the germline mutation *DMAP1* c.907−5C>G, and in individuals My6621 and My6660 with missense variants in *DMAP1*, we did not detect second hits in the tumors (Table 2). However, capillary sequencing does not detect e.g., structural and epigenetic alterations. In our previous study,^14^ we reported a few ULs with mutations in genes encoding SRCAP complex subunits without observed second hits. Despite this, in gene expression analysis, these tumors tended to cluster with other SRCAP complex mutated ULs and display decreased expression of the mutated gene encoding an SRCAP subunit. We cannot rule out the possibility that in particular the two missense variants are not causative, although reduced H2A.Z levels and low MAFs of variant alleles suggest pathogenic nature of the mutations. In addition, the pathogenicity of the *DMAP1* c.907−5C>G mutation was strongly supported by cDNA sequencing, as retention of intron 7 was seen at the transcript level.

Exceptionally, one of the studied *YEATS4* germline mutated ULs (My6606m6) showed bi-allelic hypermethylation, thus harboring three separate hits at *YEATS4* (Figure 3). Three separate hits were also detected in one UL (My6638m1) with the predisposing *DMAP1* mutation and two somatic mutations (Table 2). The three-hit hypothesis assumes that the mutated allele of a tumor suppressor may retain some residual function, and although bi-allelic mutation is sufficient to initiate tumorigenesis, selection during tumor progression might favor cells with more complete inactivation. The three-hit model has shown to be applicable in *APC* in colorectal cancer,^50^^,^^51^ and it can be speculated whether this same model could be applicable for *YEATS4* and *DMAP1* in case of mutations with possible residual activity.

The tumors of the seven individuals with inherited variants in genes encoding SRCAP complex subunits in our own sample collection were frequently cellular ULs (Table 3), and the majority of ULs were intramurally located. Clinical features of individuals with inactivating *FH* germline mutations causing HLRCC include early age of onset, familial aggregation, and multiplicity of ULs.^52^^,^^53^ Our analysis of 19,208 individuals with ULs showed that individuals with a germline LoF mutation affecting the SRCAP complex displayed a significantly earlier age at UL diagnosis (mean 43.2 years) than the remaining individuals with ULs (mean 48.8 years). In our in-house material, the seven individuals with a variant in a gene encoding SRCAP complex subunits were 28–47 years old at the time of diagnosis and the age at hysterectomy ranged from 38 to 52 years. Three of the individuals had one solitary UL and four had multiple ULs. The size of the largest UL in each individual varied from a diameter of 32–110 mm. Four individuals with a variant in a gene encoding SRCAP complex subunits showed familial aggregation of ULs and five had family history of hysterectomies (Table 3). These clinical features suggest that ULs with an inherited mutation affecting the SRCAP complex mimic the UL phenotype seen in individuals with inherited *FH* deficiency: multiple and/or large tumors at relatively young age with a family history of ULs. Individuals with these clinical characteristics are the most appropriate candidates for genetic testing of inherited mutations in genes encoding SRCAP complex subunits as well as *FH* mutations. Cutaneous leiomyomatosis as well as renal cell cancer are additional important features suggesting *FH* mutation.^10^^,^^24^ This and our previous work^14^ have demonstrated the potential of H2A.Z IHC prescreening followed by targeted sequencing of genes encoding SRCAP complex subunits in identification of somatic and germline mutations of genes encoding SRCAP complex subunits.

Three of the top-ranking genes, *YEATS4*, *DMAP1*, and *ACTL6A*, are also involved in another H2A.Z-loading complex, Ep400/Tip60.^13^ Besides these three genes, no other members of the Ep400/Tip60 complex displayed association to UL in our analysis. Note that *ZNHIT1* is specific to the SRCAP complex; we have previously shown that somatic mutations in *ZNHIT1* can lead to deficient H2A.Z deposition in tumors, suggesting that Ep400/Tip60 cannot rescue the loss of SRCAP complex function.^14^ Given the outstanding role *ZNHIT1* had in our analysis here, our results provide further evidence for the specific link between SRCAP complex defects and UL. Why all nine genes encoding SRCAP complex subunits are not associated with UL is an intriguing question to be addressed in further studies. Furthermore, work toward understanding the basis of the remarkably tissue-specific effect of germline and somatic mutation affecting the SRCAP complex could shed important light on the mechanisms of UL genesis in general.

The large-scale UKB exome-sequencing cohort enabled us to define a focused set of candidate genes for the germline screening of our own sample collection of individuals with ULs. This approach was indeed very successful, and we were able to identify causative inherited defects in the associated genes encoding SRCAP complex subunits. Identification of predisposing genes associated with development of ULs enables targeted testing of family members, active family planning, and follow-up of the individuals. It has been shown that the molecular subclass of the UL has an influence on the treatment response,^54^ so the genetic background of the tumor may have implications for the new management and prevention strategies tailored to an individual’s genetic defect.

## Data Availability

The biobank datasets used during this study are available at UK Biobank (https://www.ukbiobank.ac.uk/) and at the AstraZeneca portal (https://azphewas.com/). NGS data have been archived at the European Genome-phenome Archive (EGA) under accession number EGAD00010001924. The Nanopore sequencing data are accessible in the manuscript and EGA under accession number EGAD00010001925. The requests for the data at EGA should be addressed to the data access committee of two University of Helsinki representatives independent from the authors of this study (dac-finlandmyomastudy@helsinki.fi). This study did not generate code.

## Supplemental information

Document S1. Figures S1–S4, Tables S1, S2, S4, and S5

Table S3. Phenome-wide associations of loss-of-function variants in genes encoding SRCAP complex subunits The nine genes encoding SRCAP complex subunits were tested for loss-of-function (LoF) variant associations against 15,500 binary phenotypes. A putative significance threshold was chosen at p < 0.0005 to account for multiple testing over the nine genes encoding SRCAP complex subunits and 15,500 binary phenotypes. The table gives summary statistics for a gene-based collapsing test, including the gene symbol, phenotype, variant model (details in Material and methods), category of the phenotype, p value (two-sided Fisher’s exact test; details in Material and methods) and numbers of individuals with the phenotype and numbers of controls. Numbers of qualifying variant (QV) are also given. OR: odds ratio; LCI and UCI: lower and upper 95% confidence intervals, respectively

Document S2. Article plus supplemental information
