# Supplementary material for: Inherited mutations affecting the SRCAP complex are central in moderate-penetrance predisposition to uterine leiomyomas
Source: Am J Hum Genet. 2023 Feb 10;110(3):460–74. doi: 10.1016/j.ajhg.2023.01.009 (PMC10027472; doi:10.1016/j.ajhg.2023.01.009)
Supplement: Document S1. Figures S1–S4, Tables S1, S2, S4, and S5 [file mmc1.pdf]

The American Journal of Human Genetics, Volume *110*

Supplemental information

Inherited mutations affecting the SRCAP complex are central in moderate-penetrance predisposition to uterine leiomyomas

Niko Välimäki, Vilja Jokinen, Tatiana Cajuso, Heli Kuisma, Aurora Taira, Olivia Dagnaud, Sini Ilves, Jaana Kaukomaa, Annukka Pasanen, Kimmo Palin, Oskari Heikinheimo, Ralf Bützow, Lauri A. Aaltonen, and Auli Karhu

SUPPLEMENTAL FIGURES

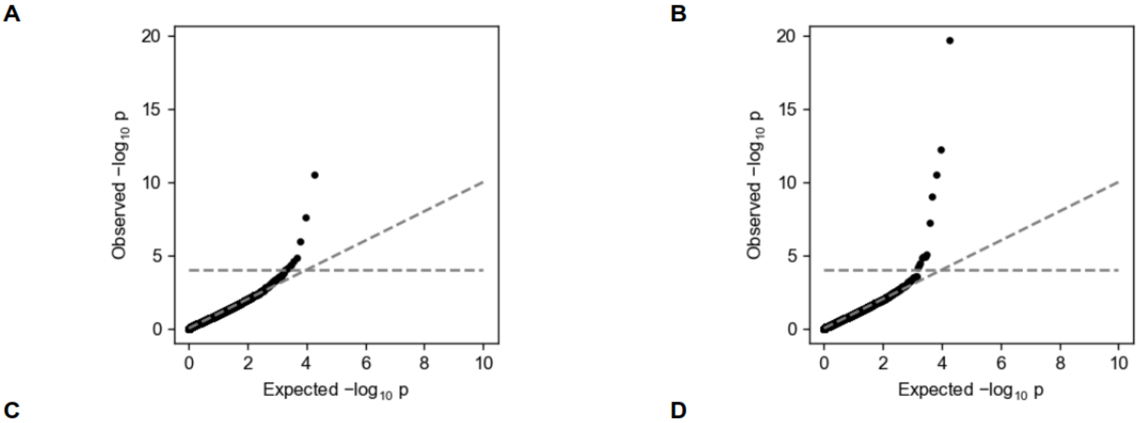

C

Gene	Number of variants [a]	Ultra-rare variants [b]	MAC [c]	MAC [d] (controls)	Rank	OR	p
MSH6	4	101	140	127	1	3.3	1.9e-20
YEATS4 [e]	2	13	55	33	2	5.0	5.7e-13
ZNHIT1 [e]	3	9	54	61	3	2.6	3.0e-11
BRCA1	4	94	120	188	4	1.9	9.5e-10
CHEK2	9	40	452	990	5	1.4	6.4e-8
MSH2	0	31	30	29	6	3.1	9.2e-6
NAE1	0	33	35	40	7	2.6	1.2e-5
DMAP1 [e]	4	22	68	115	8	1.8	1.2e-5
FH	1	12	24	20	9	3.6	1.5e-5
MLH1	2	22	37	62	10	1.8	3.4e-5
GREB1	2	64	54	78	11	2.1	5.0e-5
ACAT2	1	22	18	55	12	1.0	7.0e-5
…							
ACTL6A [e]	0	8	9	4	21	6.7	4.8e-4
ACTR6	1	26	16	54	14,414	0.9	0.8
SRCAP	1	13	7	20	14,892	1.0	0.8
RUVBL2	0	6	3	9	17,113	1.0	1.0
VPS72	1	19	21	58	17,903	1.1	1.0
RUVBL1	0	2	0	2	n/a	n/a	n/a

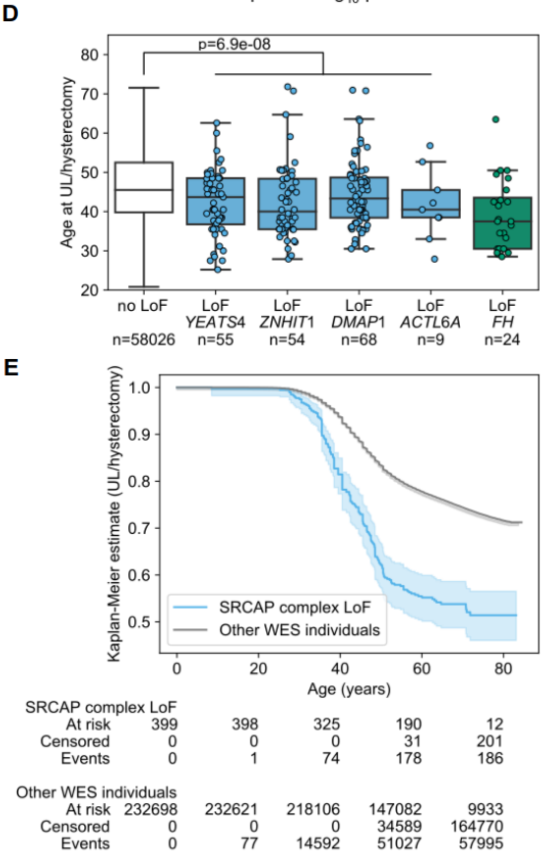

Figure S1: **Germline loss-of-function variant associations to individuals with uterine leiomyoma or hysterectomy operation.** A-B, quantile-quantile plots of SKAT-O test p values across all 18,899 genes: A, uterine leiomyoma (UL) phenotype, and B, the combined phenotype of UL or hysterectomy. The diagonal dashed line shows where the observed and expected null distributions match, and the horizontal dashed line shows p=1e-4. C-E, summary statistics for

58,709 individuals with UL or hysterectomy and 174,905 female controls. Genes that passed exome-wide significance at p<1e-4 are shown, including a summary of all nine SRCAP complex genes. The p values are from gene-based SKAT-O tests. Rank: rank among 18,899 genes; OR: odds ratio; MAC: minor allele count for loss-of-function (LoF) variants; [a] Number of LoF variants with minor allele frequency≤1%, excluding ultra-rare variants; [b] Number of ultra-rare LoF variants with MAC≤10; [c] MAC among individuals with UL/hysterectomy; [d] MAC among female controls; [e] SRCAP complex genes that passed p<0.05/9; n/a: no statistics were computed for MAC<3 genes. D-E**,** *YEATS4*, *ZNHIT1*, *DMAP1* and *ACTL6A* LoF variants contribute to younger age at UL diagnosis or hysterectomy (p=6.9e-8; two-sided Welch's t test). D, age at UL diagnosis or hysterectomy, stratified by the gene with a LoF variant. Box plots show the median and the first and third quartiles of the data; whiskers extend up to 1.5 interquartile range (IQR) and dots show the individual observations. *FH* is shown as a reference. No LoF: individuals without LoF variants among the genes highlighted. E, Kaplan-Meier estimates (p=3.8e-31; two-sided log-rank test) and at risk, censored and event counts for the five time points on the X-axis. Individuals with *FH* LoF variants were excluded from the Kaplan-Meier estimates.

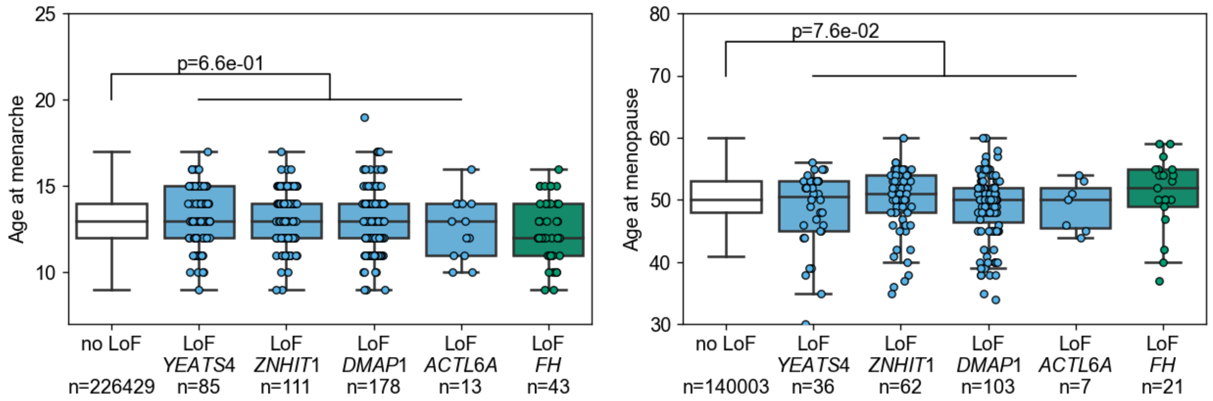

Figure S2. No differences in age at menarche or menopause were observed for women with loss-of-function of SRCAP complex genes. *YEATS4*, *ZNHIT1*, *DMAP1* and *ACTL6A* were examined for loss-of-function (LoF) variant association to age at menarche and menopause. Box plots stratified by the gene with a LoF variant, showing the median and the first and third quartiles of the data; whiskers extend up to 1.5 IQR. Coloured dots show the individual observations with LoF variants. *FH* is shown as a reference. On the left, age at menarche stratified by LoF status. No difference was observed (p=0.66; two-sided Welch's t test). On the right, age at menopause stratified by the gene with a LoF variant. No difference was observed (p=0.076; two-sided Welch's t test). Altogether 226,859 and 140,232 women had age at menarche and menopause information available, respectively. No LoF: women with no LoF variants among the genes highlighted.

A

Gene	Variant category	Number of QV (individuals with UL)	Number of QV (controls)	OR	p
YEATS4 [a]	ptv5pcnt/ptv	23	58	4.96	1.4e-08
DMAP1 [a]	ptv5pcnt/ptv	22	74	3.72	1.6e-06
YEATS4 [a]	ptvraredmg/flexdmg	23	94	3.06	1.5e-05
TMED7	UR	11	23	5.98	2.1e-05
ZNHIT1 [a]	ptv5pcnt/ptv	21	84	3.13	2.6e-05
SRPK2	flexdmg	44	264	2.09	2.8e-05
ZNF697	rec	7	8	10.94	4.6e-05
MMP11	raredmg	38	218	2.18	5.9e-05
MMP11	ptvraredmg	42	259	2.03	9.1e-05
DNASE2	rec	9	18	6.25	9.2e-05
ZNHIT1 [a]	ptvraredmg/flexdmg	32	178	2.25	9.9e-05

B

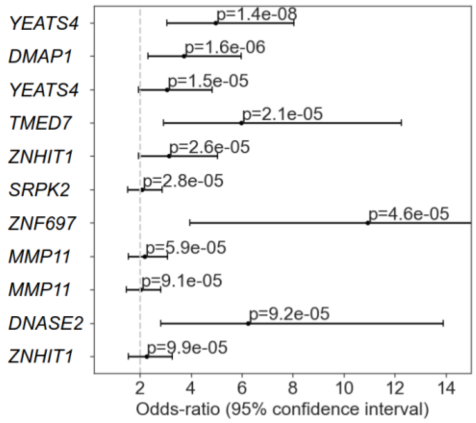

Figure S3. Summary of moderate-penetrance associations to uterine leiomyoma. A uterine leiomyoma endpoint comprising 15,780 individuals with ULs and 197,159 female controls was inspected for gene-level associations across ten different variant categories and 19,000 genes. Altogether eleven associations displayed a moderate effect size (gene-based collapsing test, odds-ratio [OR]>2.0 and p value<1e-4). A, details for numbers qualifying variants (QV), odds-ratios (OR) and p values (Fisher's exact test). ptv5pcnt/ptv: Protein-truncating variants (MAF≤5% had the same variants as MAF≤0.1%). ptvraredmg, flexdmg, raredmg: rare non-synonymous, predicted damaging variants (MAF≤0.025%, MAF≤0.1%, MAF≤0.025%, respectively). UR: ultra-rare damaging variants (MAF≤0.005%). rec: recessive variant model (MAF≤1%). [a] SRCAP complex member genes. B, odds ratios, p values and 95% confidence intervals. The dashed-line shows the OR>2.0 cutoff.

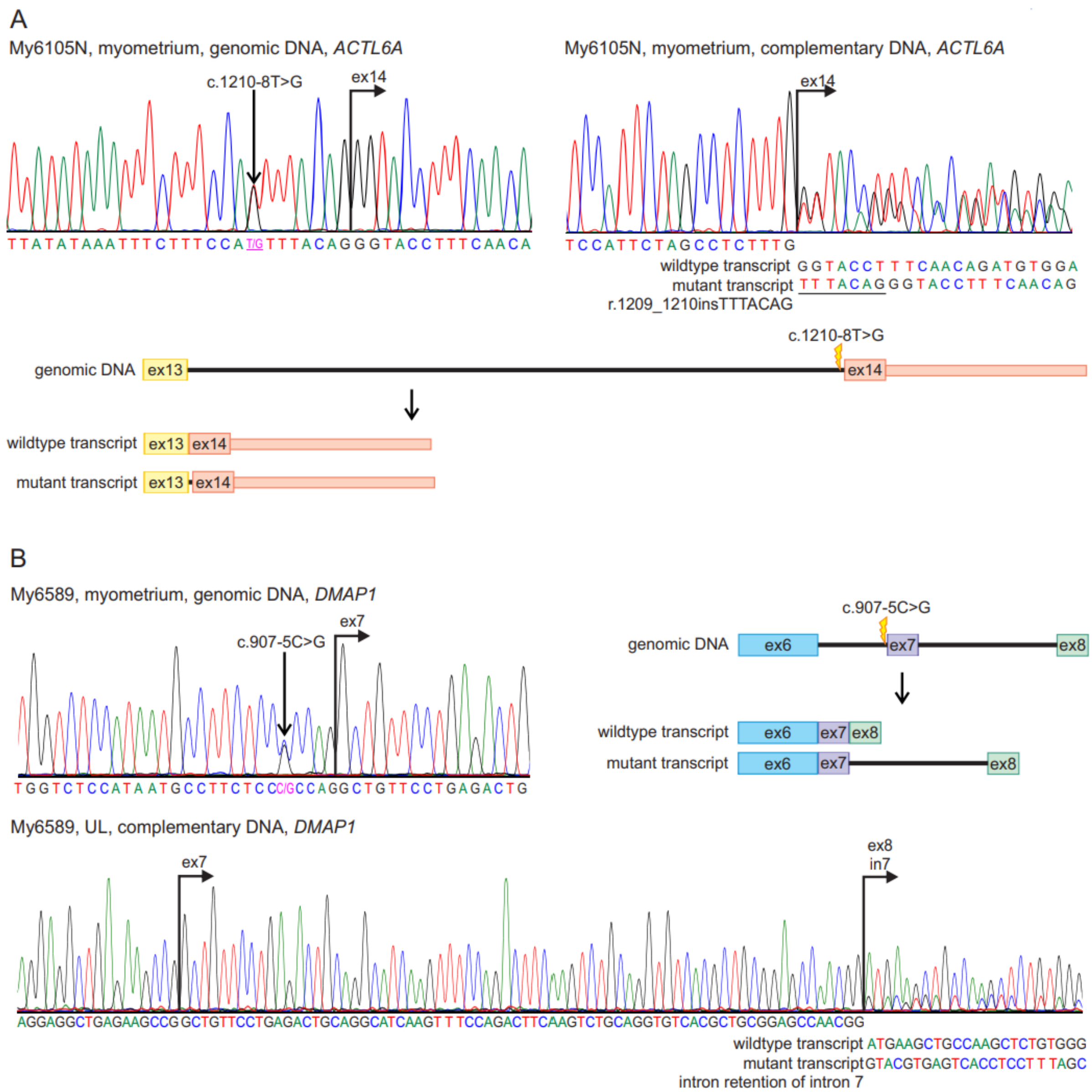

Figure S4. Splice-site variants of SRCAP complex members in two individuals with ULs. A) Sanger sequencing of genomic DNA from normal myometrium confirmed that individual My6105 has an intronic germline variant c.1210-8T>G in *ACTL6A*. Sequencing of complementary DNA (cDNA) revealed that this mutation creates a new splice site, resulting in a 7bp insertion c.1209_1210insTTTACAG at the transcript level, breaking the reading frame. B) Individual My6589 harbored a germline mutation *DMAP1* c.907-5C>G. At the transcript level, cDNA sequencing revealed intron retention of intron 7.

SUPPLEMENTAL TABLES

Table S1. Primers used for the mutation screening.

Gene and exon(s)	Primer sequences 5'-3'	Amplicon length
ACTL6A exon 1	GGCTATCGCTCCTCGAGAC<br>CGGTTACAAACCCCACACG	172 bp
ACTL6A exons 2-3	TATTTGGAAGACTGAGGCGG<br>TGTCTGGAAATCAGTTTGTGG	680bp
ACTL6A exon 4	TTACATTTGGGACAGGTCTGG<br>CCATTAGGGTGTTTTGTTGTTC	560bp
ACTL6A exon 5	TGAGATATTATGAAAGATGATTTCCC<br>GGAACAAGCAATAATTGGCG	345bp
ACTL6A exon 6	GAGCCTGCCCTTTACAATAAAC<br>TCAACACATAAATAAAGCCATGC	355bp
ACTL6A exons 7-8	TCACATAAATACACCCACAGAGG<br>CCATATTTTACTCAAGGATTTACAGC	477bp
ACTL6A exons 9-10	AAACATGGTCCAATTTCATTCAG<br>CAAAACATTTTAGCCACAAAAGC	550bp
ACTL6A exon 11	GTGGCTAAAATGTTTTGGATATG<br>ATGATTTGGGGACATTTCAGTC	221bp
ACTL6A exon 12	CCAGGATGGAGAATTGATGG<br>ACCAGGCACACACAAAATATC	382bp
ACTL6A exon 13	GCGAGGAAGACCCTGTCTC<br>AGGTCTTCCACTCATTTGACAG	318bp
ACTL6A exon 14	GGTTGTAAGCTACAATCACGCC<br>AAAGATGGTCATTCTTTTCCTG	302bp
DMAP1 exon 1	TGAGCCTGGTCCTTCTTCAG<br>AGTGTAGCACCCATCCCTTG	261bp
DMAP1 exon 2	GCTTTGTTCAGGGATTGAGC<br>AACTAGGAGTTAGGGGCATGG	247bp
DMAP1 exon 3	AGGGTAGGGTAGATCAGCTCC<br>AGCTCGCACAAATGGCTC	350bp
DMAP1 exons 4-5	GTCACTGGGGCCTGGAG<br>AGTGGGAGTTGAGGGGATG	602bp
DMAP1 exons 6-7	TCACCTCCGTGTCTACCCTC<br>CTGCTCCATCCCCACCTG	582bp
DMAP1 exon 8	TTCCACCATCTTTCCCTCTG<br>CCTGCCCAACCTATATCCAC	226bp
DMAP1 exon 9	GAGGGGAGTTCACATTGCTG<br>CACTAGAGGGAGAGGAGCCC	451bp
DMAP1 exon 10	TGAAGCACATGCACTAAGCC<br>GAAAGGAAAAGGAAGCAGCC	210bp
YEATS4 exon 1	CCAAGTAACTCGCCCTCCTT<br>GAGAAAAGGCGCGAAAGGAA	291bp

YEATS4 exon 2	AGCCTGCCATTCTTTAAAGCA<br>ACAGGTTGTCTTTAAGCAAAACA	290bp
YEATS4 exons 3-4	ACTTCCCAGGTGTAGTTCATGT<br>AGGATTTTGAAAGGCGCTACA	581bp
YEATS4 exons 5-6	TGGGTGATTTGCTGCAATAAGT<br>GGCACGAATCATACAACCCT	496bp
YEATS4 exons 7	CATTGTCGTCAGGAAATGCC<br>CCATTTCTCCAGTGAAGCCC	328bp
ZNHIT1 exon 1	ACGCGCAGAAGTACAAGCTA<br>GCGAAAGAGCGAGACCAAAA	220bp
ZNHIT1 exon 2	TGGGGATGAGATCAGAGAGC<br>GTCCTCCCACAGCCTGAGT	373bp
ZNHIT1 exon 3	TGAACCAGAGAAAGCTGCTG<br>GCCGGCTCTAGAACTCCTC	246bp
ZNHIT1 exon 4	GTTGGAGGAGCAGGTGAGAG<br>AGGATGAGGGGAGAGAGGTC	373bp
ZNHIT1 exon 5	CTGTCTGTGGCTTCCCATCC<br>CAGAATCTCTCGCGATCAGGG	585bp
**Primers to validate splice effect of the *ACTL6A* c.1210-8T>G germline mutation**		
Forward primer aligning to exon 12	GCAGTGTAATAGTGGCAGGAG	wildtype 336bp<br>mutated 343bp
Reverse primer aligning to exon 14	AGATGGTCATTCTTTTCCTGAGT	
**Primers to validate splice effect of the *DMAP1* c.907-5C>G germline mutation**		
Forward primer aligning to exon 5	GCTTGAGCGTCTCTACAACC	wildtype 342bp<br>mutated 670bp
Reverse primer aligning to exon 8	TGTTCCAGGGCCTTGATCTT	

Table S2. *MSH6* and *BRCA1* hysterectomy associations arose with endometrial and ovarian cancer. Summary numbers of individuals with loss-of-function (LoF) variants stratified by hysterectomy and cancer endpoints. Among individuals with *MSH6* and *BRCA1* LoF germline mutations, hysterectomy operations were explained to a large degree by endometrial and ovarian cancer, respectively. Breast cancer and *CHEK2* are shown for reference. Odds ratios (OR) and p values are from a two-sided Fisher's exact test.

		**No hysterectomy**		**Hysterectomy operation**			
**Gene**	**Phenotype**	**No cancer**	**Individuals with cancer**	**No cancer**	**Individuals with cancer**	**OR**	**p**
MSH6	Breast cancer	114	13	126	13	0.9	8.4E-01
MSH6	Endometrial cancer	127	0	89	50	n/a [a]	6.0E-17
MSH6	Ovarian cancer	127	0	125	14	n/a [a]	1.0E-04
MSH6	Malignant neoplasms of female genital organs	123	4	71	68	29.5	3.7E-19
BRCA1	Breast cancer	133	55	69	51	1.8	2.0E-02
BRCA1	Endometrial cancer	185	3	113	7	3.8	5.1E-02
BRCA1	Ovarian cancer	184	4	91	29	14.7	1.2E-09
BRCA1	Malignant neoplasms of female genital organs	178	10	79	41	9.2	6.9E-11
CHEK2	Breast cancer	826	160	356	93	1.3	4.4E-02
CHEK2	Endometrial cancer	985	1	427	22	50.7	9.0E-11
CHEK2	Ovarian cancer	982	4	430	19	10.8	4.5E-07
CHEK2	Malignant neoplasms of female genital organs	948	38	395	54	3.4	2.1E-08

[a] n/a: no odds ratio estimates available.

Table S3. Phenome-wide associations of loss-of-function variants in SRCAP complex genes. The nine SRCAP complex genes were tested for loss-of-function (LoF) variant associations against 15,500 binary phenotypes. A putative significance threshold was chosen at $p<0.0005$ to account for multiple testing over the nine SRCAP complex genes and 15,500 binary phenotypes. The table gives summary statistics for a gene-based collapsing test, including the gene symbol, phenotype, variant model (details in Material and methods), category of the phenotype, p value (two-sided Fisher's exact test; details in Material and methods) and numbers of individuals with the phenotype and numbers of controls. Numbers of qualifying variant (QV) are also given. OR: odds ratio; LCI and UCI: lower and upper 95% confidence intervals, respectively.

Table S3 is provided as a separate Excel file.

Table S4. Results of H2A.Z prescreening IHC. Uterine leiomyoma (UL) sections were stained together with the *YEATS4* and *MED12* mutated ULs used as negative and positive controls, respectively. Samples were classified based on the immunoreaction intensity into three groups: 0 = negative or weak, 1 = moderate, 2= strong.

Individual ID	Tumor	H2A.Z score
**My6451**	My6451m1	2
**My6458**	My6458m1	1
**My6463**	My6463m1	1
	My6463m2	2
**My6499**	My6499m1	2
**My6512**	My6512m1	2
**My6517**	My6517m1	2
**My6519**	My6519m1	2
**My6527**	My6527m1	2
**My6533**	My6533m1	2
**My6542**	My6542m1	2
**My6544**	My6544m1	2
**My6547**	My6547m1	2
**My6564**	My6564m1	1
**My6569**	My6569m1	2
**My6571**	My6571m1	2
**My6576**	My6576m1	2
**My6589**	My6589m1	1
**My6593**	My6593m1	2
**My6601**	My6601m1	2
**My6604**	My6604m1	2
**My6605**	My6605m1	2
	My6605m2	0
	My6605m3	2
**My6615**	My6615m1	2
**My6621**	My6621m1	1
**My6623**	My6623m1	2
**My6624**	My6624m1	2

**My6626**	My6626m1	2
**My6634**	My6634m3	2
**My6635**	My6635m5	2
**My6636**	My6636m1	2
**My6638**	My6638m1	1
	My6638m2	1
	My6638m3	0
**My6640**	My6640m1	2
**My6644**	My6644m1	2
**My6645**	My6645m2	2
	My6645m6	2
**My6652**	My6652m2	2
**My6655**	My6655m1	2
**My6660**	My6660m1	1
**My6661**	My6661m1	1
**My6665**	My6665m1	1
**My6667**	My6667m3	2
**My6668**	My6668m1	2
	My6668m4	2
**My6673**	My6673m3	2
**My6674**	My6674m1	2
**My6675**	My6675m4	2
	My6675m7	0
**My6676**	My6676m4	2
**My6677**	My6677m1	0
**My6679**	My6679m4	2
**My6680**	My6680m1	2
**My6687**	My6687m1	2
**My6692**	My6692m1	2
**My6696**	My6696m1	1
**My6701**	My6701m1	0

Table S5. Reduction of H2A.Z levels in germline mutated *ACTL6A*, *YEATS4*, and *DMAP1* ULs. Individual My6105 had a germline *ACTL6A* mutation, and individuals My6564 and My6606 shared a germline mutation in *YEATS4*. Four individuals (My6638, My6589, My6621, My6660) harbored *DMAP1* germline mutations. Samples are classified based on the immunoreaction intensity into three groups: 0 = negative/weak, 1 = moderate, 2= strong.

Individual ID	Tumor	H2A.Z score
**My6105**	My6105m1	0
	My6105m4	0
	My6105m5	0
**My6564**	My6564m1	1
**My6606** [a]	MH19-A	1
	MH19-B	1
	MH19-C	0
	MH19-D	0
	MH19-E	0
	MH19-F	0
	MH19-H	0
	MH19-I	1
	MH19-K	0
	MH19-L	0
	MH19-M	0
**My6589**	My6589m1	1
**My6621**	My6621m1	1
**My6638**	My6638m1	1
	My6638m2	1
	My6638m3	0
**My6660**	My6660m1	1

[a] formalin fixed paraffin embedded blocks of this individual from diagnostic laboratory
